# Genetic and phenotypic characterization of *NKX6‐2*‐related spastic ataxia and hypomyelination

**DOI:** 10.1111/ene.14082

**Published:** 2019-10-17

**Authors:** V. Chelban, M. Alsagob, K. Kloth, A. Chirita‐Emandi, J. Vandrovcova, R. Maroofian, I. Davagnanam, S. Bakhtiari, M. D. AlSayed, Z. Rahbeeni, H. AlZaidan, N. T. Malintan, J. Johannsen, S. Efthymiou, E. Ghayoor Karimiani, K. Mankad, S. A. Al‐Shahrani, M. Beiraghi Toosi, M. AlShammari, S. Groppa, N. A. Haridy, L. AlQuait, A. Qari, R. Huma, M. A. Salih, R. Almass, F. B. Almutairi, M. H. Hamad, I. A. Alorainy, K. Ramzan, F. Imtiaz, M. Puiu, M. C. Kruer, T. Bierhals, N. W. Wood, D. Colak, H. Houlden, N. Kaya

**Affiliations:** ^1^ Department of Neuromuscular Diseases University College London Institute of Neurology London UK; ^2^ Department of Neurology and Neurosurgery Institute of Emergency Medicine Chisinau Moldova; ^3^ Department of Genetics KFSHRC Riyadh Saudi Arabia; ^4^ Institute of Human Genetics University Hospital Hamburg‐Eppendorf Hamburg Germany; ^5^ Genetics Department University ‘Victor Babes’ Timisoara Romania; ^6^ Genetics Research Centre, Molecular and Clinical Sciences Institute St George's University of London London UK; ^7^ Brain Repair and Rehabilitation University College London Institute of Neurology London UK; ^8^ Barrow Neurological Institute Phoenix Children's Hospital Phoenix AZ USA; ^9^ Department of Child Health, Cellular and Molecular Medicine Department of Neurology University of Arizona College of Medicine Phoenix Phoenix AZ USA; ^10^ Medical Genetics KFSHRC Riyadh Saudi Arabia; ^11^ Clinical and Experimental Epilepsy University College London Institute of Neurology London UK; ^12^ Department of Paediatrics University Hospital Hamburg‐Eppendorf Hamburg Germany; ^13^ Great Ormond Street Hospitals London UK; ^14^ Department of Paediatric Diseases Faculty of Medicine Mashhad University of Medical Sciences Mashhad Iran; ^15^ Department of Neurology and Psychiatry Assiut University Hospital Assiut Egypt; ^16^ Neurology Division, Department of Pediatrics College of Medicine, King Saud University KFSHRC Riyadh Saudi Arabia; ^17^ Department of Radiology & Medical Imaging College of Medicine, King Saud University KFSHRC Riyadh Saudi Arabia; ^18^ Department of Biostatistics, Epidemiology and Scientific Computing KFSHRC Riyadh Saudi Arabia

**Keywords:** hypomyelination, leukodystrophy, NKX6‐2, spastic ataxia 8, SPAX8

## Abstract

**Background and purpose:**

Hypomyelinating leukodystrophies are a heterogeneous group of genetic disorders with a wide spectrum of phenotypes and a high rate of genetically unsolved cases. Bi‐allelic mutations in *NKX6‐2* were recently linked to spastic ataxia 8 with hypomyelinating leukodystrophy.

**Methods:**

Using a combination of homozygosity mapping, exome sequencing, and detailed clinical and neuroimaging assessment a series of new *NKX6‐2* mutations in a multicentre setting is described. Then, all reported *NKX6‐2* mutations and those identified in this study were combined and an in‐depth analysis of *NKX6‐2*‐related disease spectrum was provided.

**Results:**

Eleven new cases from eight families of different ethnic backgrounds carrying compound heterozygous and homozygous pathogenic variants in *NKX6‐2* were identified, evidencing a high *NKX6‐2* mutation burden in the hypomyelinating leukodystrophy disease spectrum. Our data reveal a phenotype spectrum with neonatal onset, global psychomotor delay and worse prognosis at the severe end and a childhood onset with mainly motor phenotype at the milder end. The phenotypic and neuroimaging expression in *NKX6‐2* is described and it is shown that phenotypes with epilepsy in the absence of overt hypomyelination and diffuse hypomyelination without seizures can occur.

**Conclusions:**

*NKX6‐2* mutations should be considered in patients with autosomal recessive, very early onset of nystagmus, cerebellar ataxia with hypotonia that rapidly progresses to spasticity, particularly when associated with neuroimaging signs of hypomyelination. Therefore, it is recommended that *NXK6‐2* should be included in hypomyelinating leukodystrophy and spastic ataxia diagnostic panels.

## Introduction

Hypomyelinating leukodystrophies are a heterogeneous group of genetic disorders with a wide spectrum of phenotypes. Given that myelination is a highly regulated process these disorders usually result from genetic abnormalities. However, the majority of individuals with hypomyelinating disorders have no genetic diagnosis 1.

Hypomyelination can result from dysfunctions in myelin generation or maintenance pathways including mutations in the myelin proteins (*PLP1*), protein translation (*POLR3A*,* POLR3B*,* POLR1C*) and gap junction proteins linking astrocytes and oligodendrocytes (*GJC2*).

A new phenotype associated with bi‐allelic mutations in *NKX6‐2* leading to spastic ataxia 8 (SPAX8), autosomal recessive, with hypomyelinating leukodystrophy (OMIM: 617560) has recently been described by our group 2. The reported *NKX6‐2* mutations were bi‐allelic truncating or located in the highly conserved homeobox domain. Clinically, they presented with early onset spastic ataxia or hypotonia progressing to severe spasticity within a few months and were associated with hypomyelination 2.

Here, a large, ethnically diverse cohort is presented, describing comprehensively an expanding clinical and neuroimaging syndrome and a large genotypic spectrum of *NKX6‐2*‐related disease.

## Methods

The study included affected individuals with spastic ataxia and hypomyelination from unrelated families of different ethnic backgrounds. Families were recruited under Institutional Review Board/ethics‐approved research protocols (UCLH: 04/N034) with informed consent. For comprehensive genotype–phenotype analyses, all reported genetically diagnosed *NKX6‐2* mutations were included. Extended methods are given in Appendix S1.

## Results

### Genotype spectrum in *NKX6‐2*‐related disease

**Figure 1:**
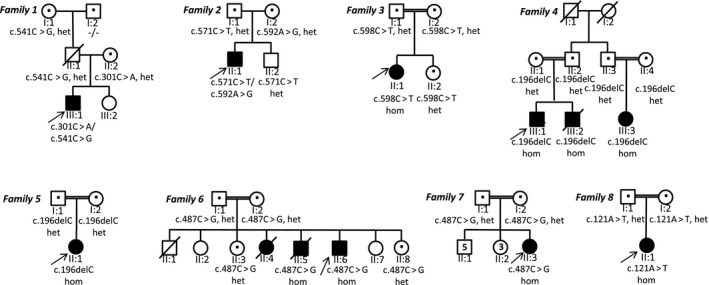
Family trees in all new families reported in this study. het, heterozygous; hom, homozygous; the individuals tested in this study are indicated with a dot.

**Figure 2:**
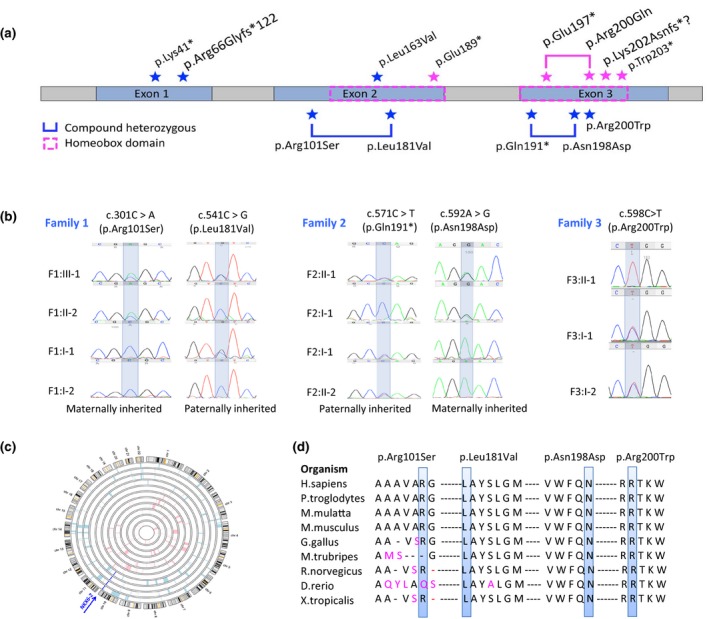
Mutation spectrum of *NKX6‐2*‐related disease. (a) *NKX6‐2* gene with all the mutations identified. All known and novel mutations identified in our cohort are labelled with a blue star; all mutations previously reported are labelled with a magenta star and plotted on top of the gene. (b) Sanger sequencing confirmation with segregation analysis for novel *NKX6‐2* variants reported in this study. (c) Homozygosity mapping in family IV and V identified a homozygous region on chromosome 10, shared by affected individuals and containing the pathogenic homozygous variant c.196delC in *NKX6‐2*. (d) Conservation across species of each novel missense mutation reported in this study.

In this study, 11 new cases from eight families (Fig. 1) carrying pathogenic variants in *NKX6‐2* were identified. Eight distinct mutations were found including four novel variants (Fig. 2a). One was present in gnomAD with very low allele frequency in the heterozygous state (MAF 0.0001170) but absent as homozygous (c.541C>G) (Fig. 2b), and three were known pathogenic variants. The c.196delC identified in families IV and V was present in a shared homozygous region (Fig. 2c). All missense variants were located in conserved amino acid positions (Fig. 2d).

Our analysis was extended to include 33 individuals from 21 families carrying *NKX6‐2* mutations identified in this study and previously reported 2, 3, 4, 5 (Table 1, Appendices S2 and S3). So far, 13 distinct *NKX6‐2* variants have been linked to SPAX8 disease. Several mutations (c.121A>T, c.487C>G, c.196delC) were reported in multiple families. The c.196delC and c.487C>G were identified in five families each, all originally from the Middle East. Haplotype analysis from three families confirmed that c.196delC was a founder mutation. However, the c.487C>G carriers did not share the same haplotype, the mutation arising recurrently 5. The c.121A>T was identified in three families of Indian origin. Haplotype analysis data from two of these families confirmed a founder effect 2.

**Table 1:** Variant description of all *NXK6‐2* mutations reported to date

Zygosity	cDNA change	Amino acid change	Type of mutation	Novel/known	ACMG score	ACMG classification	Onset	Phenotype	Additional signs reported	Hypomyelination	Cerebellar atrophy	Reference
Compound heterozygous	c.301C>A	p.Arg101Ser	Missense	Novel	PM2, PM3, PP1, PP3	Likely pathogenic	Childhood	Predominantly motor delay	Seizures	Mild	Yes	This study
	c.541C>G	p.Leu181Val	Missense	Known (rs369901030) (MAF = 0.0001170)	PM1, PM3, PP1, PP3	Likely pathogenic						This study
Compound heterozygous	c.571C>T	p.Gln191*	Nonsense	Novel	PVS1, PM1, PM2, PM3, PP1	Pathogenic	Neonatal	Severe global psychomotor delay	No	Diffuse, severe	No	This study
	c.592A>G	p.Asn198Asp	Missense	Novel	PM1, PM2, PM3, PP1	Likely pathogenic						This study
Homozygous	c.598C>T	p.Arg200Trp	Missense	Novel	PS1, PM1, PM2, PM3, PP1	Pathogenic	Neonatal	Severe global psychomotor delay	No	Diffuse, severe	Yes	This study
Homozygous	c.121A>T	p.Lys41*	Nonsense	Known	PVS1, PS3, PM2, PM3, PP1	Pathogenic	Childhood	Predominantly motor delay	Dystonia. Limitation of eye movements	Diffuse, severe	Yes	This study, Chelban *et al*. 2
Homozygous	c.196delC	p.Arg66Glyfs*122	Frameshift	Known	PVS1, PM2, PM3, PP1	Pathogenic	Neonatal	Severe global psychomotor delay	Limitation of eye movements, hearing impairment. Gastrostomy for severe dysphagia. Scoliosis	Diffuse, severe	No	This study, Anazi *et al*. 4, Baldi *et al*. 5
Homozygous	c.487C>G	p.Leu163Val	Missense	Known	PM1, PM2, PM3, PP1	Likely pathogenic	Neonatal	Severe global psychomotor delay	Seizures. Gastrostomy for severe dysphagia	Variable. 2 cases reported with no hypomyelination	Yes	This study, Chelban *et al*. 2, Baldi *et al*. 5
Homozygous	c.565G>T	p.Glu189*	Nonsense	Known	PVS1, PM1, PM2, PM3, PP1	Pathogenic	Neonatal	Severe global psychomotor delay	Severe dystonia	Diffuse, severe	Yes	Dorboz *et al*. 3
Compound heterozygous	c.589C>T	p.Gln197*	Nonsense	Known	PVS1, PM1, PM2, PM3, PP1	Pathogenic	Neonatal	Severe global psychomotor delay	Swallowing difficulties. Poor visual acuity	Diffuse, severe	No	Dorboz *et al*. 3
	c.599G>A	p.Arg200Gln	Missense	Known	PM1, PM2, PM3, PP1	Likely pathogenic						
Homozygous	c.606delinsTA	p.Lys202Asnfs?1	Frameshift	Known	PVS1, PM1, PM2, PM3, PP1	Pathogenic	Neonatal	Severe global psychomotor delay	Severe dystonia	Diffuse, severe	Yes	Dorboz *et al*. 3
Homozygous	c.608G>A	p.Trp203*	Nonsense	Known	PVS1, PM1, PM2, PM3, PP1	Pathogenic	Neonatal	Severe global psychomotor delay	Seizures	Diffuse, severe	No	Baldi *et al*. 5

ACMG, The American College of Medical Genetics and Genomics.

**Figure 3:**
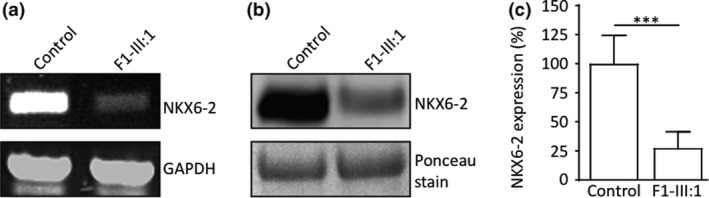
Functional analyses and pathogenicity of variants identified in family 1. (a) Reverse transcription polymerase chain reaction in compound heterozygous missense case F1‐III:1 showed absent or severely reduced *NKX6‐2* compared to control; glyceraldehyde 3‐phosphate dehydrogenase (GAPDH) was used as a loading control. (b) Reduced NKX6‐2 protein levels confirmed by western blot in individual F1‐III:1. Total protein lysate extracted from human fibroblasts assessed by sodium dodecyl sulfate polyacrylamide gel electrophoresis and analysed by western blotting using anti‐NKX6‐2 antibody (left panel). (c) Densitometry analysis shows significant reduction in NKX6‐2 protein levels in fibroblasts harbouring the *NKX6‐2* missense mutation compared to control cells. ***P < 0.01, replicate values, mean and SD are shown; one‐way anova with Bonferroni *post hoc* test.

With one exception (p.Arg101Ser), all missense mutations affected the homeobox domain. To establish the deleterious effect of p.Arg101Ser variant, reverse transcription polymerase chain reaction (RT‐PCR) and western blot were performed. Control RT‐PCR for glyceraldehyde 3‐phosphate dehydrogenase (GAPDH) confirmed the presence of cDNA in all samples. In the patient, *NKX6‐2* cDNA was severely reduced compared to controls (Fig. 3a). Immunoblot analysis confirmed a significant reduction in NKX6‐2 protein levels in the patient compared to controls (Fig. 3b, c, Appendix S4).

The majority of reported cases (81.8%, 27/33) presented in the first year of life, half of these (14/27) as neonates. Whether the mutation type influenced the age of onset was analysed. Sixteen cases carried two truncating alleles, 13 had bi‐allelic missense mutations and four had compound heterozygous variants including one truncating mutation. Although an earlier mean onset age was observed in the group harbouring two truncating alleles versus the group with two missense alleles (7.3 months vs. 8.3 months), the difference was not statistically significant (*P* = 0.78).

### Phenotype spectrum in *NKX6‐2*‐related disease

Assessment of the age of onset and disease severity revealed two ends of an expanding phenotype spectrum of *NKX6‐2* mutations.

#### Neonatal and very early onset

**Figure 4:**
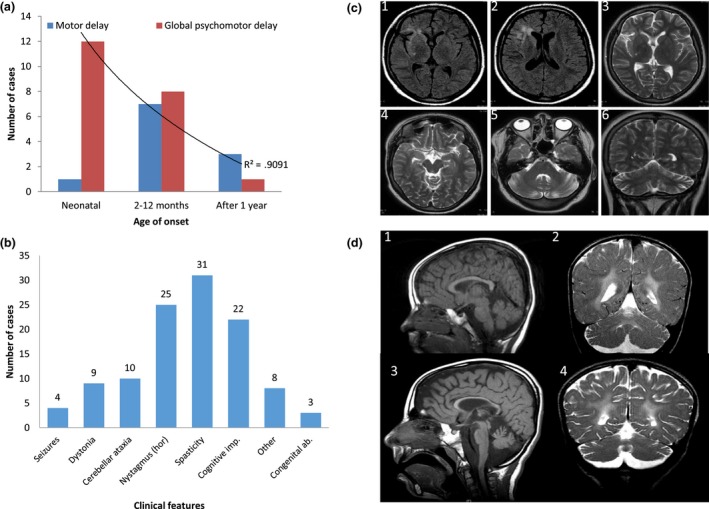
Genetype–phenotype correlation and neuroimaging spectrum of *NKX6‐2* mutations. (a) The neonatal‐onset group has a statistically significant higher frequency of global psychomotor developmental delay (red) compared with the other two groups (onset from 2 months to 1 year and onset after 1 year). Childhood onset is associated with predominantly motor delay (blue). *P* = 0.05, *r* ^2^ = 0.9. (b) Clinical features associated with *NKX6‐2* mutations (the horizontal axis) with the number of cases on the vertical axis (total *n* = 33). hor, horizontal gaze‐evoked nystagmus. (c) Fluid‐attenuated inversion recovery and T2‐weighted MRI acquisitions from case F1‐III:1 exhibiting T2 hyperintense signal change in periventricular WM surrounding the frontal horn of the right lateral ventricle, and frontal and temporal opercular and subinsular WM T2‐weighted hyperintense signal change associated with a degree of cortical volume loss. Note the normal signal intensity of the globi pallidi, thalami and external capsules, mesencephalon and pons. There is disproportionate cerebellar volume loss with mild T2‐weighted hyperintense signal change in the peri‐dentate WM. (d) Longitudinal MRI in case F7‐II:3 at ages of 4 years (D1, D2) and 8½ years (D3, D4) (D1, D3, mid‐sagittal T1‐weighted; D2, D4, coronal T2‐weighted) showing progressive thinning of the corpus callosum and cerebellar atrophy associated with WM abnormality sparing the U fibres (2, 4). There is progressive enlargement of the cortical sulci and extra axial cerebrospinal fluid spaces indicating underlying global brain atrophy.

Neonatal onset was associated with a higher rate of severe global psychomotor disability compared with onset after 1 month of age (*P* = 0.05) (Fig. 4a). Twenty cases with information on motor milestones showed very severe motor deficit, all children failing to achieve independent ambulation and 70% (14/20) failing to achieve head control. Furthermore, 90% of children with disease onset before 1 year never achieved verbal milestones/meaningful speech.

Complex medical needs included severe dysphagia requiring gastrostomy (6/33 cases), congenital heart disease (1/33), respiratory failure (2/33) leading to death, undescended testicles (1/33), severe dental and/or gum abnormalities (6/33 cases) and inguinal hernia (1/33). Survival in this group was shorter with 12.1% (4/33) of children dying in their first 5 years of life.

#### Childhood onset

Childhood onset with predominantly motor delay and complex spastic ataxia was identified in seven individuals in this study and those previously reported 2. Case F1‐III:1 has the least severe motor phenotype in our series (Appendix S5). Case F8‐II:1 has a phenotype resembling the previously reported cases with the c.121A>T mutation – severe spastic ataxia but relatively preserved cognition 2. The seven cases all achieved ambulation, although they required walking aids (walking frame) 1–3 years into the disease and wheelchair 3–8 years later. Features including head titubation and severe dystonia in the previously reported patients who achieved adulthood suggest that they may be related to disease progression rather than genotype.

#### Complex spastic ataxia and developmental delay

The most common symptom at onset was nystagmus (25/33 cases) (Fig. 4b) described as horizontal gaze‐evoked in the majority of cases. Other ocular manifestations were square wave jerks, hypometric saccades, impaired smooth pursuit and reduced visual acuities (four cases). Limitation of bilateral, lateral gaze eye movements was seen later in three cases who reached adulthood.

Spasticity with brisk reflexes in the upper and lower limbs and upgoing plantar responses were present in all cases. Axial hypotonia was reported at presentation in nine patients, all with onset of disease before 1 year of age associated with upper and lower limb spasticity soon after presentation. Cervical and/or limb dystonia was present in around 40% of cases where information was available. Examination of the peripheral nervous system was normal in all cases and normal nerve conduction studies were reported in two cases who reached adulthood.

Seizures were present in four (12.5%) patients harbouring *NKX6‐2* mutations. Case F1‐III:1 presented primary tonic progressing to secondarily generalized seizure at 6 years. Electroencephalography at the age of 13 showed loss of age‐based background activity and absent anterior–posterior gradient of background activity with focal tonic seizure presenting clinically as hemifacial seizures. There were intermittent frequency slowing (most pronounced at frontal electrodes), multifocal epileptic discharges (sharp waves and sharp‐slow waves) pronounced over the right hemisphere, and several focal tonic seizures. The seizures were multidrug‐resistant. No details regarding seizure phenomenology are available in the other three cases 3, 5.

Cognitive function varied greatly between patients. Interestingly, two cases reported here (F1‐III:1 and F8‐II:1) and four previously reported 2 (total 6/33) had normal cognitive development for their age. Case F8‐II:1 and the four previously reported cases with normal cognitive development carry the homozygous nonsense mutation p.Lys41* whilst F1‐III:1 carries two missense compound heterozygous variants (p.Arg101Ser, p.Leu181Val). However, severe cognitive impairment with arrested speech development was present in 66% of all children with bi‐allelic *NKX6‐2* pathogenic variants and in all reported children with neonatal onset. It is acknowledged that, in most cases, an accurate cognitive function assessment was difficult due to severe motor impairment and/or developmental language delay.

Other features present in SPAX8 patients included strabismus (4/33 patients), scoliosis (6/33), neck or/and limb dystonia (9/33), contractures (4/33 patients), dysmorphism (2/33), hip dislocation (2/33) and single cases of hearing impairment and hirsutism.

### Neuroimaging spectrum in *NKX6‐2*‐related disease

The key neuroimaging feature in the majority of cases was a hypomyelinating leukodystrophy. Magnetic resonance imaging (MRI) (Appendix S6) showed signal abnormality with atrophy noted supratentorially within the thalami and the globus pallidi. Infratentorially, there was notable involvement of the pons with signal abnormality involving the transverse pontine fibres with relative expansion to the entire pons. This contrasted with the orthogonal oriented fibres of the corticospinal tracts of relatively normal signal, providing a distinctive prominent appearance of the mid‐pons on the axial T2‐weighted sequences. Furthermore, signal change was noted in the cerebellar hemispheres, particularly involving the subcortical white matter (WM) and the dentate nuclei. Cerebellar volume was relatively increased in very young patients, probably related to the underlying WM changes, and demonstrated mild atrophic change over time. Not all children developed cerebellar atrophy (Appendix S7).

Interestingly, in case F1‐III:1 with two missense compound heterozygous mutations neuroimaging findings were milder compared to cases carrying homozygous truncation mutations (Fig. 4c). Marked cerebellar atrophy was a key finding in this case. Furthermore, two other paediatric *NKX6‐2*‐related cases were reported previously without overt hypomyelination 4. Thinning of the corpus callosum was present in 6/33 patients. A longitudinal MRI study in F7‐II:3 at 4 and 8 years old shows progressive thinning of the corpus callosum and cerebellar atrophy, associated with WM abnormality sparing the U fibres (Fig. 4d).

## Discussion

Recently the first cases of *NKX6‐2* mutations leading to the hypomyelination and spastic ataxia phenotype in humans were described 2. Given the rarity of hypomyelinating disorders and the absence of an unbiased cohort to screen, it is difficult to estimate the frequency of *NKX6‐2* mutations. However, genetic analysis of the University College London (UCL) leukodystrophies cohort identified 10 cases of hypomyelination with pathogenic mutations in *PLP1* (four families) and single cases of *TUBB4A*,* POLR3A/B* and *CLCN2* 6. Here are presented six additional cases (three families) of hypomyelination due to *NKX6‐2* mutations from the same research group suggesting a significant burden of *NKX6‐2* mutations.

In this study, the phenotypic spectrum was expanded and it was shown that *NKX6‐2* mutations lead to a neonatal onset at the severe end and childhood onset at the milder end. Interestingly, the compound heterozygous missense variants c.301C>A (p.Arg101Ser) and c.541C>G (p.Leu181Val) led to a significant reduction (>70%) of the NKX6‐2 protein. It is possible that the small amount of NKX6‐2 protein identified by western blot provided a degree of myelination leading to a less severe clinical picture. This individual and the three cases previously published 5 presented with multidrug‐resistant epilepsy. In focal cortical dysplasia, a common cause of drug‐resistant epilepsy, hypomyelination abnormality was confirmed in numerous histopathological epilepsy surgery specimen studies 7. Focal dysplasia is currently linked to abnormalities in the differentiation of glial cells from their progenitors and their migration to the cortical place 8, a process regulated by transcription factors including *NKX6‐2* 9.

Additional clinical features associated with *NKX6‐2* mutations – cervical and/or limb dystonia, congenital abnormalities (congenital heart disease, undescended testes), severe dental and/or gum abnormalities – reflect the developmental role of *NKX6‐2* as a member of the homeobox gene family 10. These genes are involved in development, specifying geographical orientation of the body by directing the formation of limbs and organs 11. Some homeobox genes such as *PAX6* 12, *OTX2* 13 and *MEOX1* 14 are involved in a variety of developmental and neurological disorders with brain abnormalities.

Clinical and neuroimaging findings in *NKX6‐2*‐related disease reflect the involvement of WM and pyramidal tracts (spasticity, brisk reflexes, upgoing plantars), cerebellum (truncal and limb ataxia, nystagmus) and bulbar function (dysarthria, dysphagia). Complications related to these clinical manifestations led to significant impairment in vital functions including swallowing (although dysphagia was not routinely assessed in all cases, severe dysphagia requiring gastrostomy was reported in several cases), respiration and functionally disabling spasticity, similarly to other myelin‐related diseases 15. Early screening and recognition of these disease‐related complications are important aspects during the clinical assessments of these patients.

Furthermore, our study expands the MRI spectrum of *NKX6‐2* mutations from diffuse hypomyelination to focal T2‐weighted hyperintensity and parenchymal volume loss. Recently, other hypomyelinating leukodystrophy genes such as *TUBB4A* and *POLR3A* have been shown to have distinct phenotypes and neuroimaging spectrum, ranging from spastic paraplegia to spastic ataxia without overt hypomyelination to hypomyelinating leukodystrophies resulting from different mutations 16, 17. A similar pattern was identified in *NKX6‐2* with cases presenting clinically with spastic ataxia in the absence of hypomyelination and a reduction of NKX6‐2 protein levels (case F1‐III:1) compared to extended hypomyelination in cases with truncating mutations resulting in no expression of NKX6‐2 protein, as previously reported 2. Therefore, the importance of molecular diagnosis with additional functional work and confirmatory evidence is highlighted.

In conclusion, it is shown that the phenotypic and neuroimaging expression in *NKX6‐2* mutations can range from a complex, neonatal onset at the severe end and a childhood onset at the milder end of the spectrum and that phenotypes with epilepsy in the absence of overt hypomyelination, and diffuse hypomyelination without seizures, can occur. It is recommended that *NXK6‐2* should be included in hypomyelinating leukodystrophy and spastic ataxia diagnostic panels.

## Acknowledgements

The authors would like to thank the patients and their families for their essential help with this work. The authors are grateful to the UK HSP Society. This study was supported by the Spastic Paraplegia Foundation, the Medical Research Council (MRC UK MR/J004758/1, G0802760, G1001253), the Wellcome Trust in equipment and strategic award (Synaptopathies) funding (WT093205MA and WT104033/Z/14/Z), Ataxia UK, the British Neurological Surveillance Unit (BNSU) and the National Institute for Health Research (NIHR). The Sequencing and Genotyping Core Facilities at KFSHRC are also thanked for their technical help. N.K. is supported by KACST Grant (14‐MED2007‐20) and KFSHRC seed grant (RAC#2120022). I.D. receives support from the NIHR UCL/UCLH Biomedical Research Centre. Supported in part by Doris Duke Clinical Scientist Development Award 2014112 MCK and NIH NINDS NS083739 (MCK). M.A.S. was supported by the Deanship of Scientific Research, King Saud University, Riyadh, Saudi Arabia, through the research group project number RGP‐VPP‐301.

## Disclosure of conflicts of interest

The authors declare no financial or other conflicts of interest.

## Supporting information


**Appendix S1.** Extended methods.

**Appendix S2.** Genotype–phenotype description of all *NKX6‐2* mutations reported to date. Legend: NA‐not available, m‐months, y‐years, VEP‐visual evoked potentials, ERG‐electroretinogram, BAEP = brainstem auditory evoked potential, PDA = Patent Ductus Arteriosus.

**Appendix S3.** Pathogenicity and novelty assessment of all *NKX6‐2* variants identified in this study.

**Appendix S4.** Western blot analysis in individual F1‐III:1. Experiments performed three times and three lysates from case and controls are shown on the blot.

**Appendix S5.** Genotype–phenotype description of the eight new families reported in this study.

**Appendix S6.** Hypomyelination in *NKX6‐2*‐related disease. From left to right: multiple T1‐weighted (column 1) and T2‐weighted (columns 2–5) MRI acquisitions through four cases (top to bottom rows: F4‐III:1, F3‐II:1, F6‐II:6, F2‐II:1). Normal to hyperintense T1 white matter (WM) signal (column 1) in areas corresponding to the T2‐weighted hyperintense signal (column 2) confirmed hypomyelination. Column 2 demonstrates diffuse T2‐weighted hyperintense signal change in subcortical, deep WM including external capsules, globi pallidi and thalami. Columns 3 and 4 demonstrate dorsal mesencephalic and diffuse pontine T2‐weighted hyperintense signal change. Column 5 demonstrates diffuse cerebellar WM T2‐weighted hyperintense signal change including the peri‐dentate WM with relative preservation of cerebellar volume.

**Appendix S7.** Neuroimaging spectrum of *NKX6‐2*‐related disease.
