# Supplementary material for: Genetic and phenotypic characterization of NKX6‐2‐related spastic ataxia and hypomyelination
Source: Eur J Neurol. 2019 Oct 17;27(2):334–42. doi: 10.1111/ene.14082 (PMC6946857; doi:10.1111/ene.14082)
Supplement: Supplementary file 1 — Appendix S1. Extended methods. [file ENE-27-334-s001.docx]

**Supplementary S1. Methods**

**Genetic analysis**

Whole exome sequencing (WES) was performed in the probands and unaffected parents in all families as previously described [1]. Alignment was performed using BWA (<http://bio-bwa.sourceforge.net/>) [2] with GRCH37 as a reference. Variant calling, and annotation were done as outlined here [3]. Called variants were filtered for rare or novel, coding/splicing, homozygous and compound heterozygous variants using custom R scripts. All disease causing variants reported in Human Gene Mutation Database (HGMD), ClinVar [4] and variants with a minor allele frequency (MAF)<1% in 1000 Genomes Project [5], NHLBI GO Exome Sequencing [6], and genome Aggregation Database (gnomAD) [7] were included. Variants were assessed for novelty and pathogenicity. Novel variants were assessed using the American College of Medical Genetics and Genomics (ACGM) score and classification for mutations [8]. All *NKX6-2* variants (NM_177400) considered disease causing were validated with bidirectional Sanger sequencing as described previously [9]. Primer sets are:

| **c.DNA change** | **Forward primer** | **Reverse primer** |
| --- | --- | --- |
| c.301C>A | GCATCAGCGACATCCTGG | GCGCCCATGGACACTAAC |
| c.541C>G | AACCTTCGAGCAGACCAAGTAC | CGAGGGTTTGTGCTTCTTGAG |
| c.571C>T | AACCTTCGAGCAGACCAAGTAC | CGAGGGTTTGTGCTTCTTGAG |
| c.592A>G | AACCTTCGAGCAGACCAAGTAC | CGAGGGTTTGTGCTTCTTGAG |
| c.598C>T | AACCTTCGAGCAGACCAAGTAC | CGAGGGTTTGTGCTTCTTGAG |

**RT‐PCR**

For RT‐PCR analysis, total RNA was extracted from fibroblasts using RNeasy Mini Kit (Qiagen) as per manufacturers instructions. We extracted RNA from case F1-III:1 and age-matched controls, and reverse transcribed to cDNA using the High Capacity cDNA Synthesis kit (Applied Biosystems). A PCR was then performed with the following primers; Forward: GATGAAGACGTCGCTGTTCC, Reverse: CTGGAACCAGACCTTCACCT.

**Western blotting**

Fibroblast samples from individual F1-III:1 and healthy controls were obtained. After reaching 90% confluency, the cultured cells were harvested. Total protein lysates were extracted using RIPA buffer with protease inhibitors-EDTA free (Thermo Fisher Scientific). Total protein concentration was determined by Bradford assay. Equal amounts of 30 μg of protein were analysed by SDS-PAGE followed by Western blotting using anti-NKX6-2 antibody (Abcam ab179532; 1:1000). The blot was visualised and densitometry quantification of NKX6-2 bands was performed using the BioRad Chemidoc imaging system (BioRad Laboratories). Experiments were performed three times.

**Phenotype**

Clinical data were collected using standardized forms including patient history, neurological examination, and neuroimaging evaluations for each patient carrying *NKX6-2* pathogenic variants.

**Statistical analysis**

Quantitative values were reported as mean with standard deviation. To determine predictors for disease severity and age of onset we compared phenotype measures across the groups with logistic regression analysis. Statistical analyses were performed using one-way ANOVA with Bonferroni post-hoc test. P-value of ≤ 0.05 was considered statistically significant.

1. Hempel, M., et al., *De Novo Mutations in CHAMP1 Cause Intellectual Disability with Severe Speech Impairment.* Am J Hum Genet, 2015. **97**(3): p. 493-500.

2. Li, H. and R. Durbin, *Fast and accurate long-read alignment with Burrows-Wheeler transform.* Bioinformatics, 2010. **26**(5): p. 589-95.

3. Chelban, V., et al., *Mutations in NKX6-2 Cause Progressive Spastic Ataxia and Hypomyelination.* Am J Hum Genet, 2017. **100**(6): p. 969-977.

4. Landrum, M.J., et al., *ClinVar: public archive of interpretations of clinically relevant variants.* Nucleic Acids Res, 2016. **44**(D1): p. D862-8.

5. Genomes Project, C., et al., *A global reference for human genetic variation.* Nature, 2015. **526**(7571): p. 68-74.

6. Shimobayashi, E. and J.P. Kapfhammer, *Increased biological activity of protein Kinase C gamma is not required in Spinocerebellar ataxia 14.* Mol Brain, 2017. **10**(1): p. 34.

7. Lek, M., et al., *Analysis of protein-coding genetic variation in 60,706 humans.* Nature, 2016. **536**(7616): p. 285-91.

8. Richards, S., et al., *Standards and guidelines for the interpretation of sequence variants: a joint consensus recommendation of the American College of Medical Genetics and Genomics and the Association for Molecular Pathology.* Genet Med, 2015. **17**(5): p. 405-24.

9. Lessel, D., et al., *Atypical Aicardi-Goutieres syndrome: is the WRN locus a modifier?* Am J Med Genet A, 2014. **164a**(10): p. 2510-3.
