# Supplementary material for: Genetic and phenotypic characterization of NKX6‐2‐related spastic ataxia and hypomyelination
Source: Eur J Neurol. 2019 Oct 17;27(2):334–42. doi: 10.1111/ene.14082 (PMC6946857; doi:10.1111/ene.14082)
Supplement: Supplementary file 2 — Appendix S2. Genotype–phenotype description of all NKX6‐2 mutations reported to date. Legend: NA‐not available, m‐months, y‐years, VEP‐visual evoked potentials, ERG‐electroretinogram, BAEP = brainstem auditory evoked potential, PDA = Patent Ductus Arteriosus. [file ENE-27-334-s002.docx]

**Supplementary S2. Genotype-phenotype description all *NKX6-2* mutations reported to date.**

| **Study** | | **This study** | | | | | | | | | | |
| --- | --- | --- | --- | --- | --- | --- | --- | --- | --- | --- | --- | --- |
| **Family** | | **I** | **II** | **III** | **IV** | | | **V** | **VI** | | **VII** | **VIII** |
| **Subject** | | F1-III:1 | F2-II:1 | F3-III:1 | F4-III:3 | F4-III:1 | F4-III:2 | F5-II:1 | F6-II:6 | F6-II:5 | F7-II:3 | F8-II:1 |
| **Mutation** | **c.DNA change** | c.301C>A; c.541C>G | c.571C>T; c.592A>G | c.598C>T | c.196delC | c.196delC | c.196delC | c.196delC | c.487C>G | c.487C>G | c.487C>G | c.121A>T |
|  | **Amino acid change** | p.Arg101Ser; p.Leu181Val | p.Gln191*; p.Asn198Asp | p.Arg200Trp | p.Arg66Glyfs*122 | p.Arg66Glyfs*122 | p.Arg66Glyfs*122 | p.Arg66Glyfs*122 | p.Leu163Val | p.Leu163Val | p.Leu163Val | p.Lys41* |
|  | **Zygozity** | Compound heterozygous | Compound heterozygous | Homozygous | Homozygous | Homozygous | Homozygous | Homozygous | Homozygous | Homozygous | Homozygous | Homozygous |
|  | **Novel/Known variant** | Novel; Known | Novel; Novel | Novel | Known | Known | Known | Known | Known | Known | Known | Known |
| **Demographic** | **Gender** | Female | Male | Female | Female | Male | Male | Female | Male | Male | Female | Female |
|  | **Age at onset** | 5y | 3m | 3m | 1m | 1m | 1m | 1m | 1m | 1m | 7m | 2y |
|  | **Disease duration until last examination** | 9y | 9y | 12y | 6y | 15y | Died at 4 years | 6y | 10y | Died age 3 years | 8y | 4y |
|  | **Consanguinity** | No | No | Yes | Yes | Yes | Yes | Yes | Yes | Yes | Yes | No |
|  | **Ethnicity** | German | Romanian | Persian | Arab | Arab | Arab | Arab | Arab | Arab | Arab | Indian |
| **General** | **Prenatal complications** | Bleeding in first trimester | No | No | Gestational diabetes | NA | NA | No | No | No | No | No |
|  | **Head circumference** | NA | Normal at last examination | Normal at last examination | Normal at last examination | Normal at last examination | Normal at last examination | Normal at last examination | Normal at last examination | -2 SD at last examination | Normal at last examination | Normal at last examination |
|  | **Dysmorphic features** | No | No | No | No | No | No | No | No | Yes | No | No |
|  | **Congenital anomalies** | No | NA | No | No | NA | NA | No | No | PDA | No | No |
|  | **Symptom at onset** | Cerebellar ataxia and dysarthria | Nystagmus (horizontal) | Nystagmus (horizontal), Cerebellar ataxia | Nystagmus | Nystagmus | Nystagmus | Nystagmus | Nystagmus | Nystagmus | Cerebellar ataxia and nystagmus | Cerebellar ataxia |
| **Neurocognitive** | **Developmental delay** | Motor | Motor | Severe global psychomotor delay | Severe global psychomotor delay | Severe global psychomotor delay | Severe global psychomotor delay | Severe global psychomotor delay | Severe global psychomotor delay | Severe global psychomotor delay | Severe global psychomotor delay | Motor |
|  | **Spasticity/Reflexes** | UL and LL / increased | UL and LL / increased | UL and LL / increased | UL and LL / increased | UL and LL / increased | UL and LL / increased | UL and LL/ increased | UL and LL / increased | UL and LL / increased | UL and LL / increased | UL < LL / increased |
|  | **Reduced muscle tone** | No | Axial hypotonia. Progressed to spasticity | No | No | Axial hypotonia. Progressed to spasticity | Axial hypotonia. Progressed to spasticity | Axial hypotonia. Progressed to spasticity | No | Axial hypotonia. Progressed to spasticity | No | No |
|  | **Cerebellar ataxia** | Yes. Limbs and gait ataxia | Yes. Limbs and gait ataxia | No walking | No | No | No | No | No | No | Yes. Limbs and gait ataxia | Yes. Limbs and gait ataxia |
|  | **Other movement disorder** | No | No | No | No | No | No | No | No | No | No | No |
|  | **Eye examination** | Normal | Nystagmus. Strabismus (divergent) | Nystagmus (rotary), pale optic discs, hypometric saccades. Strabismus (divergent) | Nystagmus | Nystagmus | Nystagmus | Nystagmus | Nystagmus | Nystagmus | Nystagmus, hypometric sacades, pale optic discs | Normal |
|  | **Seizures** | Yes, severe therapy-refractive epilepsy | No | No | No | No | No | No | No | No | No | No |
|  | **Other clinical features** | No | No | Scoliosis | No | Gastrostomy | No | No | Gastrostomy, undescended testicles, severe caries | No | No | No |
| **Milestones** | **Achieved head control** | Yes | No | No | NA | No | No | Minor control | NA | No | No | Yes |
|  | **Achieved ambulation** | 8 months | No | 8 months | No | No | No | No | No | No | 2 years | 20 months |
|  | **Uses mobility aids (from what age)** | 13 years (walker) | NA | 3 years (walker) | No | No | No | No | No | No | 2 to 3 years (walker) | No |
|  | **Wheelchair-bound/bedridden** | No | Yes | From 3 years | Yes | Yes | Yes | Yes | Yes | NA | From age 3 years | No |
|  | **Achieved meaningful speech** | Yes | No | No | No | No | No | No | No | No | No | Yes |

| **Study** | | **Chelban et al** | | | | | | **Dorboz et al** | | | | |
| --- | --- | --- | --- | --- | --- | --- | --- | --- | --- | --- | --- | --- |
| **Family** | | **VII** | | | **VIII** | **IX** | | **X** | **XI** | | **XII** | |
| **Mutation** | **c.DNA change** | c.121A>T | c.121A>T | c.121A>T | c.121A>T | c.487C>G | c.487C>G | c.606delinsTA | c.565G>T | c.565G>T | c.599G>A; c.589C>T | c.599G>A; c.589C>T |
|  | **Amino acid change** | p.Lys41* | p.Lys41* | p.Lys41* | p.Lys41* | p.Leu163Val | p.Leu163Val | p.Lys202Asnfs*? | p.Glu189* | p.Glu189* | p.Gln197*; p.Arg200Gln | p.Gln197*; p.Arg200Gln |
|  | **Zygozity** | Homozygous | Homozygous | Homozygous | Homozygous | Homozygous | Homozygous | Homozygous | Homozygous | Homozygous | Compound heterozygous | Compound heterozygous |
|  | **Novel/Known variant** | Known | Known | Known | Known | Known | Known | Known | Known | Known | Known | Known |
| **Demographic** | **Gender** | Male | Female | Male | Female | Female | Male | Male | Male | Female | Male | Male |
|  | **Age at onset** | 5 y | 3m | 6m | 12m | 1m | 1m | 2m | 2m | 1m | 3m | 6m |
|  | **Disease duration until last examination** | 18y | 27y | 7y | 43y | 6y | 4y | 10y | Died at 16.5m | 13y | 4y | 3y |
|  | **Consanguinity** | Yes | Yes | Yes | Yes | Yes | Yes | Yes | Yes | Yes | No | No |
|  | **Ethnicity** | Indian | Indian | Indian | Indian | Arab | Arab | NA | Moroccan | Moroccan | NA | NA |
| **General** | **Prenatal complications** | No | No | No | No | No | No | No | No | No | No | No |
|  | **Head circumference** | Normal at last examination | Normal at last examination | Normal at last examination | Normal at last examination | Normal at last examination | Normal at last examination | Normal at birth.  -1 SD at last examination | Normal at birth. -1 SD at last examination | Normal at birth. -1 SD at last examination | Normal at birth. Normal at last examination | Normal at birth. Normal at last examination |
|  | **Dysmorphic features** | No | No | No | No | No | No | NA | NA | NA | NA | NA |
|  | **Congenital anomalies** | No | No | No | No | No | No | NA | NA | NA | NA | NA |
|  | **Symptom at onset** | Nystagmus | Ataxia | Nystagmus | Nystagmus | Nystagmus | Nystagmus | Nystagmus (horizontal/rotatory) | Nystagmus (horizontal/rotatory) | Nystagmus (horizontal/rotatory) | Nystagmus (pendular) | Gross motor delay |
| **Neurocognitive** | **Developmental delay** | Motor | Motor | Motor | Motor | Severe global psychomotor delay | Severe global psychomotor delay | Motor | Motor | Motor | Motor | Severe global psychomotor delay |
|  | **Spasticity/Reflexes** | UL<LL / increased | UL and LL/ increased | UL<LL / increased | UL and LL / increased | UL and LL / increased | UL and LL / increased | UL and LL /NA | UL and LL /NA | UL and LL /NA | UL and LL /NA | UL and LL /NA |
|  | **Reduced muscle tone** | No | No | No | No | No | No | Axial hypotonia. Progressed to spasticity | No | Axial hypotonia. Progressed to spasticity | Axial hypotonia. Progressed to spasticity | Axial hypotonia. Progressed to spasticity |
|  | **Cerebellar ataxia** | Yes. Limbs and gait ataxia | Yes. Limbs and gait ataxia | Yes Yes. Limbs and gait ataxia | Yes. Limbs and gait ataxia | Yes. Limbs and gait ataxia | Yes. Limbs and gait ataxia | NA | NA | NA | NA | NA |
|  | **Other movement disorder** | Dystonia in the neck and upper limbs | Dystonia in the upper limbs | Dystonia | Dystonia in the upper limbs | No | No | Severe dystonia | Severe dystonia | Severe dystonia | No | No |
|  | **Eye examination** | Nystagmus (horizontal). Limitation of the eye movements | Nystagmus (horizontal). Hypometric sacades. | Nystagmus (horizontal). Hypometric sacades. | Limitation of the eye movements | Hypometric sacades, nystagmus | Hypometric sacades, nystagmus | Nystagmus (horizontal). Optic pallor. Poor visual acuity | Nystagmus (horizontal). Optic pallor. Poor visual acuity | Nystagmus (horizontal). Optic pallor. Poor visual acuity | Nystagmus (pendular). Mild optic pallor and strabismus.Poor visual acuity | NA |
|  | **Seizure** | No | No | No | No | No | No | No | No | No | No | No |
|  | **Other clinical features** | Head titubation | Head titubation | No | Head titubation | No | No | Gastrostomy (2 y). Respiratory failure and apneic episodes (2y) | Gingival hypertrophy. Gastrostomy (2 y). Hip dislocation and scoliosis. Respiratory failure and apneic episodes (1y) | Gingival hypertrophy. Hip dislocation and scoliosis | Swallowing difficulties requiring gastrostomy. | No |
| **Milestones** | **Achieved head control** | Yes | Yes | Yes | Yes | NA | No | No | No | No | No | NA |
|  | **Achieved ambulation** | 12 m | 2 years | 2 years | 13 m | NA | NA | NA | NA | NA | NA | NA |
|  | **Uses mobility aids (from what age)** | 8 years (walking stick) | 5 years (walker) | 5 years (walker) | 4 years (walker) | NA | NA | NA | NA | NA | NA | NA |
|  | **Wheelchair-bound/bedridden** | 10 years | 8 years | 8 years | 9 years | NA | NA | NA | NA | NA | NA | NA |
|  | **Achieved meaningful speech** | Yes | Yes | Yes | Yes | No | No | No | No | No | No | Few words at 2 years |

| **Study** | | **Anazi et al** | **Baldi et al** | | | | | | | | | |
| --- | --- | --- | --- | --- | --- | --- | --- | --- | --- | --- | --- | --- |
| **Family** | | **XIII** | **XIV** | **XV** | **XVI** | | **XVII** | | **XVIII** | **XIX** | | |
| **Mutation** | **c.DNA change** | c.196delC | c.196delC | c.196delC | c.196delC | | c.487C>G | | c.487C>G | c.608G>A | | |
|  | **Amino acid change** | p.Arg66Glyfs*122 | p.Arg66Glyfs*122 | p.Arg66Glyfs*122 | p.Arg66Glyfs*122 | | p.Leu163Val | | p.Leu163Val | p.Trp203* | | |
|  | **Zygozity** | Homozygous | Homozygous | Homozygous | Homozygous | | Homozygous | | Homozygous | Homozygous | | |
|  | **Novel/Known variant** | Known | Known | Known | Known | | Known | | Known | Known | | |
| **Demographic** | **Gender** | Female | Male | Female | Male | Male | Female | Male | Male | Female | Female | Female |
|  | **Age at onset** | NA | 12m | 3y | 3m | 3m | 6w | 1m | 14m | 1w | 1m | 1m |
|  | **Disease duration until last examination** | 10y | 19m | 5y | 1y | 4y | 10y | 14m | 14m | Died in the first year of life | 12m | 12m |
|  | **Consanguinity** | Yes | Yes | Yes | Yes | Yes | Yes | Yes | Yes | Yes | Yes | Yes |
|  | **Ethnicity** | Arab | Arab | Kuwait | Arab | Arab | Oman | Oman | Arab | Arab | Arab | Arab |
| **General** | **Prenatal complications** | NA | No | No | No | No | No | No | No | Pre-mature (born 35 w gestation) | Pre-mature (born 28 w gestation) | Pre-mature (born 28 w gestation) |
|  | **Head circumference** | NA | Normal at birth | Normal at last examination | NA | NA | Normal at last examination | NA | -2 SD at last examination | -2 SD at last examination | NA | NA |
|  | **Dysmorphic features** | NA | No | NA | NA | NA | Frontal bossing, bulbous nose, hypodontia | NA | No | No | Elongated face, gum hypertrophy, high arched palate, crowded teeth. | |
|  | **Congenital anomalies** | NA | No | No | No | No | No | No | No | No | No | No |
|  | **Symptom at onset** | NA | Developmental delay, axial hypotonia | Motor regression | Spasticity | Developmental delay | Nystagmus (horizontal) | Nystagmus (horizontal) | Axial hypotonia | Encephalopathy | Likely seizures (tonic limbs and uproling eyes) | |
| **Neurocognitive** | **Developmental delay** | Severe global psychomotor delay | Severe global psychomotor delay | Severe global psychomotor delay | Severe global psychomotor delay | Severe global psychomotor delay | Severe global psychomotor delay | Severe global psychomotor delay | Severe global psychomotor delay | Severe global psychomotor delay | Severe global psychomotor delay | Severe global psychomotor delay |
|  | **Spasticity/Reflexes** | NA/ NA | UL and LL / NA | UL and LL / increased | UL and LL/ increased | UL and LL / increased | UL and LL / increased | UL and LL / NA | UL and LL/ increased | UL and LL / NA | UL and LL/ increased | UL and LL / increased |
|  | **Reduced muscle tone** | Axial hypotonia | NA | Axial hypotonia | NA | NA | Axial hypotonia | Axial hypotonia | Axial hypotonia | Axial hypotonia | Axial hypotonia | Axial hypotonia |
|  | **Cerebellar ataxia** | NA | NA | NA | NA | NA | NA | NA | NA | NA | NA | NA |
|  | **Other movement disorder** | NA | NA | NA | NA | NA | NA | NA | NA | NA | Dystonia | Dystonia |
|  | **Eye examination** | Strabismus | Nystagmus (horizontal). Visual impairment | Nystagmus (horizontal). Visual impairment | Visual impairment | No | Nystagmus (horizontal). Visual impairment | Nystagmus (horizontal) | Nystagmus (horizontal) | NA | Nystagmus (horizontal) | Nystagmus (horizontal) |
|  | **Seizure** | NA | No | No | NA | NA | Yes | Yes | No | NA | Yes | No |
|  | **Other clinical features** | Scoliosis | No | Contractures | Contractures, neck dystonia | Hearing impairment | No | Contractures. Hirsutism | No | No | Scoliosis. Contractures | |
| **Milestones** | **Achieved head control** | NA | Lost from 6m | NA | NA | NA | NA | NA | NA | NA | NA | NA |
|  | **Achieved ambulation** | NA | No | NA | NA | NA | NA | NA | NA | No | No | No |
|  | **Uses mobility aids (from what age)** | NA | No | NA | NA | NA | NA | NA | NA | No | No | No |
|  | **Wheelchair-bound/bedridden** | NA | No | NA | NA | NA | NA | NA | NA | Yes | Yes | Yes |
|  | **Achieved meaningful speech** | NA | No | No | NA | NA | NA | NA | NA | NA | NA | NA |
