# Supplementary material for: Genetic and phenotypic characterization of NKX6‐2‐related spastic ataxia and hypomyelination
Source: Eur J Neurol. 2019 Oct 17;27(2):334–42. doi: 10.1111/ene.14082 (PMC6946857; doi:10.1111/ene.14082)
Supplement: Supplementary file 3 — Appendix S3. Pathogenicity and novelty assessment of all NKX6‐2 variants identified in this study. [file ENE-27-334-s003.pdf]

Supplementary S3

Chr	Start	End	Ref	Alt	Location	Gene	Type	AAChange	1000g2015aug_all	esp6500siv2_ea	esp6500siv2_aa	esp6500siv2_all	ExAC_ALL	ExAC_AFR	ExAC_AMR	ExAC_EAS
10	134598822	134598822	G	C	exonic	NKX6-2	nonsynonymous SNV	exon2:c.C541G, p.L181V	NA	0.0002	NA	0.0002	9.97E-05	0	0	0
10	134598792	134598792	G	A	exonic	NKX6-2	stopgain	exon2:c.C571T, p.Q191X	NA	NA	NA	NA	NA	NA	NA	NA
10	134599332	134599332	T	A	exonic	NKX6-2	stopgain	exon1:c.A121T, p.K41X	NA	NA	NA	NA	NA	NA	NA	NA
10	134598662	134598662	T	C	exonic	NKX6-2	nonsynonymous SNV	exon3:c.A592G, p.N198D	NA	NA	NA	NA	NA	NA	NA	NA
10	134599257	134599257	C	-	exonic	NKX6-2	frameshift deletion	exon1: c.196delC, p.Arg66Glyfs*122	NA	NA	NA	NA	NA	NA	NA	NA
10	134599152	134599152	G	T	exonic	NKX6-2	nonsynonymous SNV	exon1:c.C301A, p.R101S	NA	NA	NA	NA	NA	NA	NA	NA
10	134598876	134598876	G	C	exonic	NKX6-2	nonsynonymous SNV	exon2:c.C487G, p.L163V	NA	NA	NA	NA	NA	NA	NA	NA

ExAC_FIN	ExAC_NFE	ExAC_OTH	ExAC_SAS	Kaviar_AF	Kaviar_AC	Kaviar_AN	gnomAD_exome_ALL	gnomAD_exome_AFR	gnomAD_exome_AMR	gnomAD_exome_ASJ	gnomAD_exome_EAS	gnomAD_exome_FIN	gnomAD_exome_NFE	gnomAD_exome_OTH	gnomAD_exome_SAS	gnomAD_genome_ALL
0.0005	0.0001	0	0	7.12E-05	11	154602	0.0001	0	0.0002	0	0	0.0004	0.0001	0.0004	0	6.49E-05
NA	NA	NA	NA	NA	NA	NA	NA	NA	NA	NA	NA	NA	NA	NA	NA	NA
NA	NA	NA	NA	NA	NA	NA	NA	NA	NA	NA	NA	NA	NA	NA	NA	NA
NA	NA	NA	NA	NA	NA	NA	NA	NA	NA	NA	NA	NA	NA	NA	NA	NA
NA	NA	NA	NA	NA	NA	NA	NA	NA	NA	NA	NA	NA	NA	NA	NA	NA
NA	NA	NA	NA	NA	NA	NA	NA	NA	NA	NA	NA	NA	NA	NA	NA	NA
NA	NA	NA	NA	NA	NA	NA	NA	NA	NA	NA	NA	NA	NA	NA	NA	NA

avsnp150	SIFT_score	SIFT_converted_rankscore	SIFT_pred	Polyphen2_HDIV_score	Polyphen2_HDIV_rankscore	Polyphen2_HDIV_pred	MutationTaster_score	MutationTaster_converted_rankscore	MutationTaster_pred	PROVEAN_score	PROVEAN_converted_rankscore	PROVEAN_pred	VEST3_score	VEST3_rankscore
rs369901030	0.011	0.555	D	0.998	0.715	D	1	0.81	D	-2.48	0.54	N	0.719	0.72
NA	.	.	.	.	.	.	1	0.81	D	.	.	.	.	.
NA	.	.	.	.	.	.	1	0.81	A	.	.	.	.	.
NA	0.003	0.682	D	1	0.899	D	1	0.81	D	-4.54	0.784	D	0.526	0.567
NA	NA	NA	NA	NA	NA	NA	NA	NA	NA	NA	NA	NA	NA	NA
NA	0.798	0.031	T	0.002	0.09	B	0.998	0.441	D	0.15	0.053	N	0.071	0.062
NA	0.002	0.721	D	1	0.899	D	1	0.81	D	-2.46	0.537	N	0.759	0.753

MetaSVM_score	MetaSVM_rankscore	MetaSVM_pred	MetaLR_score	MetaLR_rankscore	MetaLR_pred	M-CAP_score	M-CAP_rankscore	M-CAP_pred	CADD_raw	CADD_raw_rankscore	CADD_phred	DANN_score	DANN_rankscore	GenoCanyon_score	GenoCanyon_score_rankscore
1.069	0.986	D	0.955	0.985	D	0.648	0.97	D	5.24	0.708	25.6	0.997	0.82	1	0.747
.	.	.	.	.	.	.	.	.	12.02	0.98	38	0.997	0.83	1	0.747
.	.	.	.	.	.	.	.	.	9.259	0.962	35	0.994	0.613	1	0.48
1.015	0.975	D	0.986	0.996	D	0.705	0.976	D	5.112	0.689	25.3	0.996	0.747	1	0.747
NA	NA	NA	NA	NA	NA	NA	NA	NA	NA	NA	NA	NA	NA	NA	NA
-0.598	0.649	T	0.454	0.787	T	0.549	0.958	D	0.943	0.184	10.33	0.973	0.328	0.746	0.233
1.063	0.984	D	0.988	0.997	D	0.833	0.987	D	4.482	0.6	24.2	0.996	0.738	0.912	0.263

integrated_fitCons_score	integrated_fitCons_score_rankscore	integrated_confidence_value	GERP++_RS	GERP++_RS_rankscore	phyloP100way_vertebrate	phyloP100way_vertebrate_rankscore	phyloP20way_mammalian	phyloP20way_mammalian_rankscore	phastCons100way_vertebrate	phastCons100way_vertebrate_rankscore	phastCons20way_mammalian
0.598	0.34	0	3.77	0.423	6.971	0.757	0.893	0.41	1	0.715	0.984
0.765	0.991	0	3.77	0.423	8.733	0.912	0.893	0.41	1	0.715	0.997
0.598	0.34	0	3.12	0.348	1.532	0.354	0.788	0.318	1	0.715	0.996
0.765	0.991	0	2.89	0.326	5.485	0.664	0.911	0.421	1	0.715	0.999
NA	NA	NA	NA	NA	NA	NA	NA	NA	NA	NA	NA
0.598	0.34	0	2.42	0.286	0.893	0.278	0.878	0.384	0.949	0.328	0.993
0.598	0.34	0	1.83	0.242	1.473	0.348	0.037	0.153	0.951	0.329	0.997

phastCons20way_mammalian_rankscore	SiPhy_29way_logOdds	SiPhy_29way_logOdds_rankscore	Interpro_domain	regsnp_fpr	regsnp_disease	regsnp_splicing_site	dpsi_max_tissue	dpsi_zscore	REVEL	MCAP
0.507	15.006	0.71		NA	NA	NA	0.1055	0.5	0.824	0.648072378
0.653	15.006	0.71	Homeobox domain	NA	NA	NA	-5.0039	-2.5	NA	NA
0.625	11.332	0.485	NA	NA	NA	NA	NA	NA	NA	NA
0.75	11.932	0.519	Homeobox domain	NA	NA	NA	NA	NA	0.937	0.704530124
NA	NA	NA	NA	NA	NA	NA	NA	NA	NA	NA
0.574	5.085	0.139	NA	NA	NA	NA	NA	NA	0.207	0.548574815
0.653	6.722	0.224	Homeobox domain	NA	NA	NA	0.0605	0.344	0.827	0.832563598

CLINSIG	CLNDBN	CLNACC	CLNDSDB	CLNDSDBID
NA	NA	NA	NA	NA
NA	NA	NA	NA	NA
Pathogenic	SPASTIC ATAXIA 8, AUTOSOMAL RECESSIVE, WITH HYPOMYELINATING LEUKODYSTROPHY	RCV000494722.1	MedGen:OMIM	CN303160:617560
NA	NA	NA	NA	NA
NA	NA	NA	NA	NA
NA	NA	NA	NA	NA
Pathogenic	SPASTIC ATAXIA 8, AUTOSOMAL RECESSIVE, WITH HYPOMYELINATING LEUKODYSTROPHY	RCV000494724.1	MedGen:OMIM	CN303160:617560
