# Supplementary figures and images for: Genetic and phenotypic characterization of NKX6‐2‐related spastic ataxia and hypomyelination
Source: Eur J Neurol. 2019 Oct 17;27(2):334–42. doi: 10.1111/ene.14082 (PMC6946857; doi:10.1111/ene.14082)

Supplemental S4

A

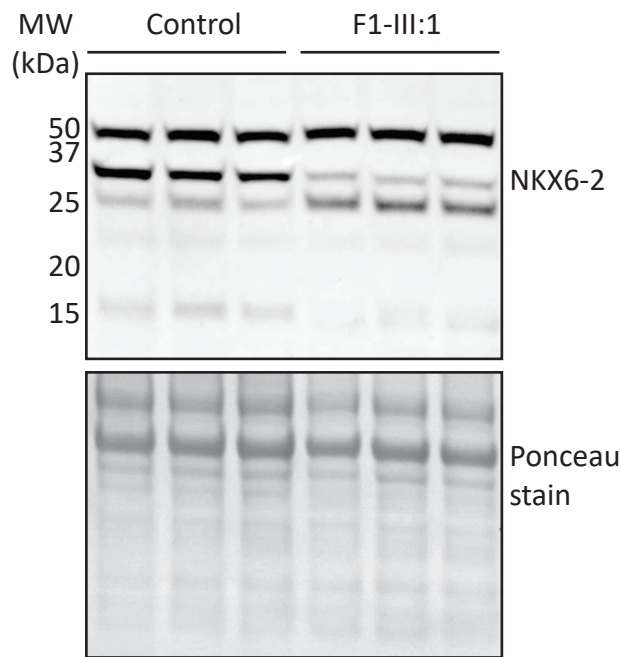

B

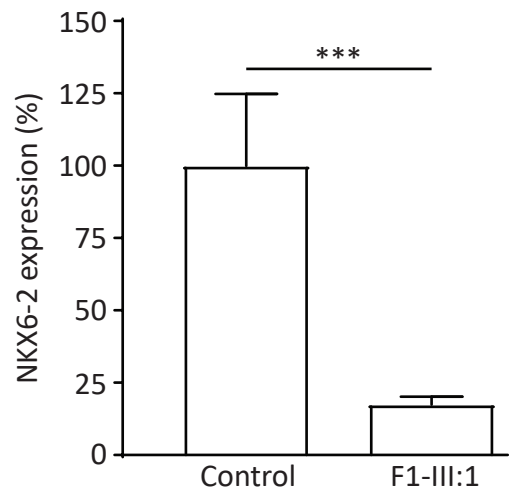

Supplement: Supplementary file 4 — Appendix S4. Western blot analysis in individual F1‐III:1. Experiments performed three times and three lysates from case and controls are shown on the blot. [file ENE-27-334-s004.pdf]

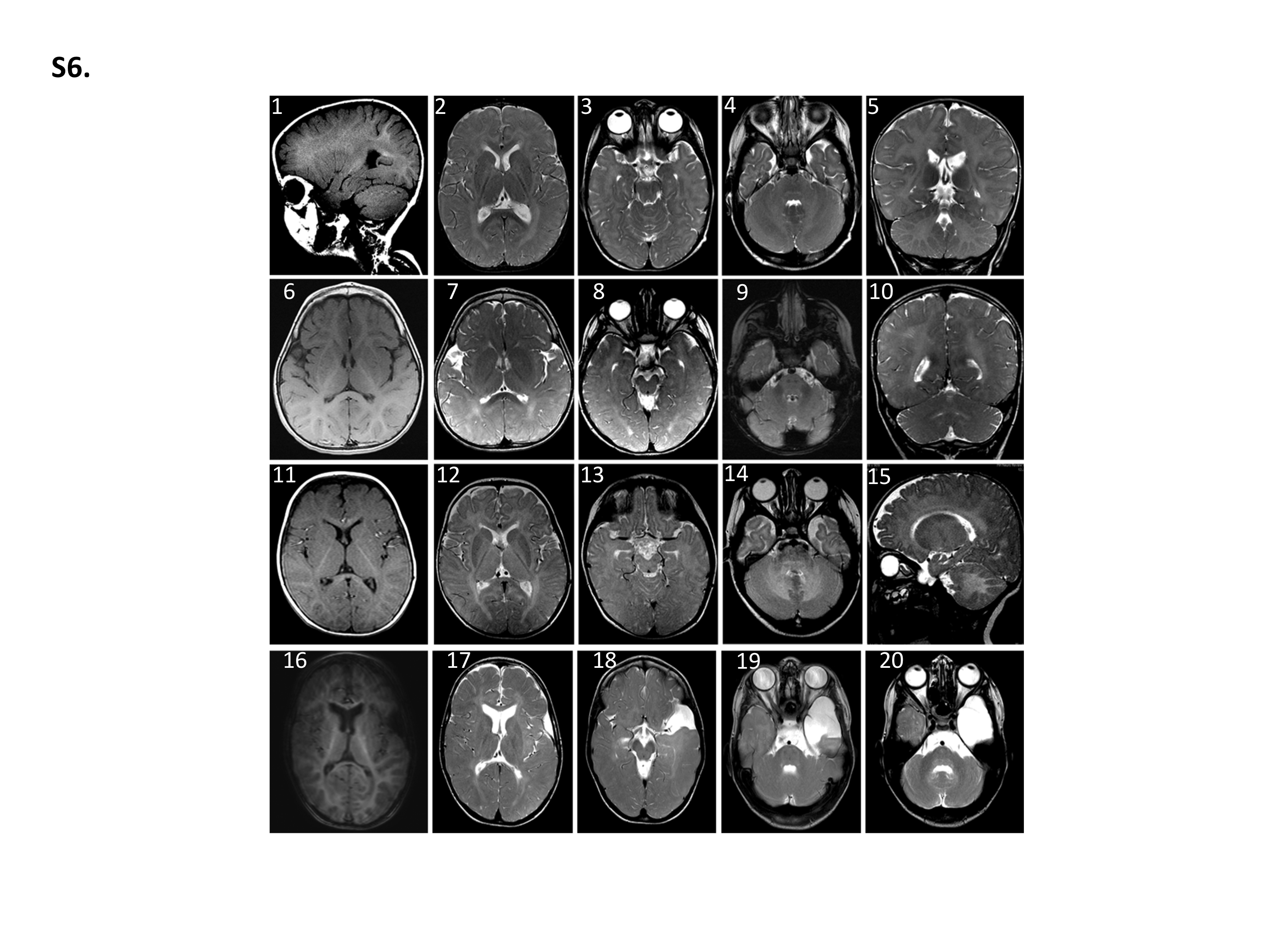

Supplement: Supplementary file 6 — Appendix S6. Hypomyelination in NKX6‐2‐related disease. From left to right: multiple T1‐weighted (column 1) and T2‐weighted (columns 2–5) MRI acquisitions through four cases (top to bottom rows: F4‐III:1, F3‐II:1, F6‐II:6, F2‐II:1). Normal to hyperintense T1 white matter (WM) signal (column 1) in areas corresponding to the T2‐weighted hyperintense signal (column 2) confirmed hypomyelination. Column 2 demonstrates diffuse T2‐weighted hyperintense signal change in subcortical, deep WM including external capsules, globi pallidi and thalami. Columns 3 and 4 demonstrate dorsal mesencephalic and diffuse pontine T2‐weighted hyperintense signal change. Column 5 demonstrates diffuse cerebellar WM T2‐weighted hyperintense signal change including the peri‐dentate WM with relative preservation of cerebellar volume. [file ENE-27-334-s006.tiff]
