# Supplementary material for: Genetic and phenotypic characterization of NKX6‐2‐related spastic ataxia and hypomyelination
Source: Eur J Neurol. 2019 Oct 17;27(2):334–42. doi: 10.1111/ene.14082 (PMC6946857; doi:10.1111/ene.14082)
Supplement: Supplementary file 5 — Appendix S5. Genotype–phenotype description of the eight new families reported in this study. [file ENE-27-334-s005.docx]

**S5. Genotype-Phenotype description of the eight new families reported in this study.**

Family I is of German descent. Early childhood and initial gross motor development of proband F1-III:1 were unremarkable. She presented with ataxia and speech development regression aged 5 years, and first tonic, secondary-generalized, seizure aged 6 years. At age 13 years neurological examination revealed spastic-ataxia and focal seizures. Whole exome sequencing performed in the proband and unaffected relatives identified two novel compound heterozygous missense variants in *NKX6-2*, c.301C>A (p.Arg101Ser) and c.541C>G (p.Leu181Val). Segregation analysis showed that p.Arg101Ser was maternally inherited and p.Leu181Val was present in the paternal grandmother confirming the bi-allelic status (Figure 2B). The p.Arg101 and p.Leu181 are conserved and highly conserved amino acids in eutherians (Figure 2C), and the two variants identified in the proband are predicted likely pathogenic and pathogenic respectively (Table 1). The p.Leu181Val had a frequency of 0.0001 in gnomAD database while p.Arg101Ser was absent from all publicly available databases and in-house ethnically matched controls.

Family II is of Romanian descent. The proband F2-II:1 presented aged 3 months with nystagmus and hypotonia that progressed to spasticity. The boy did not achieve initial motor milestones and was unable to sit-up or walk. He never achieved head control and verbal output. The initial symptoms were noticed after routine childhood immunization. The disease progressed and by age 8 years the patient was wheelchair-bound with a severe pyramidal weakness and spasticity, predominantly involving lower limb, associated with nystagmus, reduced up-gaze and ataxia. Genetic analysis revealed two novel compound heterozygous mutations in *NKX6-2,* the nonsense c.571C>T (p.Gln191*) and the missense c.592A>G (p.Asn198Asp) each carried by the unaffected parents. The c.571C>T (p.Gln191*) creates a premature stop codon predicted to cause loss of the Homeobox domain and the c.592A>G (p.Asn198Asp) is a missense located in highly conserved amino-acid position in the Homebox functional domain with a CADD score of 25.

In an Iranian, consanguineous family, patient F3-II:1 presented at 3 months with nystagmus and ataxia. She never achieved head control. At age 7 months she could say 2 words but has had no other meaningful verbal output since then. At 8 months she was able to sit up and hold objects but has had no further motor development. At last examination age 12 years she had severe pyramidal weakness, spasticity, strabismus and ataxia. We identified the c.598C>T (p.Arg200Trp) homozygous mutation in the proband, also present in heterozygous state in both parents. The c.598C>T is a novel amino acid change in a known pathogenic position located within the Homeobox functional domain.

Families IV, V, VI and VII are consanguineous from Saudi Arabia. Families IV and V share a known homozygous truncating variant in *NKX6-2*, c.196delC (p.Arg66Glyfs*122​) that fully segregated with the disease. Proband F4-III:3 had nystagmus diagnosed soon after birth. She later developed severe pyramidal weakness, spasticity, delayed motor milestones, and never achieved head control or independent ambulation. At last examination aged 6 years, she presented global developmental delay, nystagmus and severe spastic tetraplegia, bedridden with no meaningful verbal output. Two cousins were similarly affected (F4-III:1 and F4-III:2) one of whom died at four years of age. A similar clinical picture was reported in the proband from family 5 (F5-II:1) who presented with nystagmus soon after birth and progressed to global psychomotor developmental delay.

Family VI and VII carry a previously reported homozygous missense mutation c.487C>G (p.Leu163Val) with many children affected. The cases reported here (cases F6-IV:11 and F6-IV:10, F7-III:3) experienced disease onset in the neonatal period with nystagmus and hypotonia. The disease progressed to severe spastic quadriplegia and delay in both receptive and expressive language skills. The patients never achieved head control or ambulation. Furthermore, patient F6-IV:11 required gastrostomy at 3 years due to dysphagia and recurrent aspirations. His sibling, F6-IV:10 died suddenly at 3 years with no further details available.

Family VIII is of Gujarati origin. The proband (case F8-III:1) presented at 2 years with ataxia and motor developmental delay affecting mainly gross and fine motor skills. She started walking at 20 months, had normal speech and cognitive development. At last examination at 4 years she had ataxia, intention tremor, walked with a wide-base gait with normal ocular movements and normal cognitive development for age. Genetic analysis found a known homozygous nonsense mutation c.121A>T (p.Lys41*) that segregates in both unaffected parents.
