# Supplementary material for: Genetic and phenotypic characterization of NKX6‐2‐related spastic ataxia and hypomyelination
Source: Eur J Neurol. 2019 Oct 17;27(2):334–42. doi: 10.1111/ene.14082 (PMC6946857; doi:10.1111/ene.14082)
Supplement: Supplementary file 7 — Appendix S7. Neuroimaging spectrum of NKX6‐2‐related disease. [file ENE-27-334-s007.docx]

S7. Neuroimaging spectrum of *NKX6-2*-related disease.

| **Study** | | **This study** | | | | | | | | | | |
| --- | --- | --- | --- | --- | --- | --- | --- | --- | --- | --- | --- | --- |
| **Family** | | **I** | **II** | **III** | **IV** | | | **V** | **VI** | | **VII** | **VIII** |
| **Subject** | | 1 | 2 | 3 | 4 | 5 | 6 | 7 | 8 | 9 | 10 | 11 |
| **Mutation** | **c.DNA change** | c.301C>A; c.541C>G | c.571C>T; c.592A>G | c.598C>T | c.196delC | c.196delC | c.196delC | c.196delC | c.487C>G | c.487C>G | c.487C>G | c.121A>T |
|  | **Amino acid change** | p.Arg101Ser; p.Leu181Val | p.Gln191*; p.Asn198Asp | p.Arg200Trp | p.Arg66Glyfs*122 | p.Arg66Glyfs*122 | p.Arg66Glyfs*122 | p.Arg66Glyfs*122 | p.Leu163Val | p.Leu163Val | p.Leu163Val | p.Lys41* |
|  | **Zygozity** | Compound heterozygous | Compound heterozygous | Homozygous | Homozygous | Homozygous | Homozygous | Homozygous | Homozygous | Homozygous | Homozygous | Homozygous |
| **Neuroimaging** | **Hypomyelination** | No | External capsules, globi pallidi, thalami, periventricular, cerebellar | External capsules, globi pallidi, thalami, periventricular, cerebellar | External capsules, globi pallidi, thalami, periventricular, cerebellar | NA | NA | External capsules,  thalami,  periventricular | External capsules,  thalami,  periventricular | External capsules, globi pallidi, thalami, periventricular | External capsules, globi pallidi, thalami, periventricular | External capsules, globi pallidi, thalami, periventricular |
|  | **Cerebral atrophy** | No | No | Mild cerebral atrophy | No | NA | NA | No | No | No | Yes | No |
|  | **Basal ganglia abnormalities** | No | No | No | No | NA | NA | Yes | No | Yes | No | Yes |
|  | **Peri/Paraventricular cysts** | No | No | No | No | NA | NA | No | No | No | No | No |
|  | **Thin corpus callosum** | No | No | No | No | NA | NA | No | No | No | Yes | No |
|  | **Cerebellar atrophy** | Yes, vermis and superior cerebellar peduncles | No | Yes, vermis and superior cerebellar peduncles | No | NA | NA | No | No | Yes, vermis and superior cerebellar peduncles | Yes, vermis and superior cerebellar peduncles | Yes, vermis and superior cerebellar peduncles |
|  | **Other investigations** | Chronic lesion in the fronto-temporal white matter.  EEG- multifocal epileptic discharges. | No | Abnormal VEP. Normal ERG. | No | NA | NA | No | No | No | No | Normal NCS and EMG |

| **Study** | | **Chelban et al** | | | | | | **Dorboz et al** | | | | |
| --- | --- | --- | --- | --- | --- | --- | --- | --- | --- | --- | --- | --- |
| **Family** | | **VII** | | | **VIII** | **IX** | | **X** | **XI** | | **XII** | |
| **Subject** | | 12 | 13 | 14 | 15 | 16 | 17 | 18 | 19 | 20 | 21 | 22 |
| **Mutation** | **c.DNA change** | c.121A>T | c.121A>T | c.121A>T | c.121A>T | c.487C>G | c.487C>G | c.606delinsTA | c.565G>T | c.565G>T | c.599G>A; c.589C>T | c.599G>A; c.589C>T |
|  | **Amino acid change** | p.Lys41* | p.Lys41* | p.Lys41* | p.Lys41* | p.Leu163Val | p.Leu163Val | p.Lys202Asnfs*? | p.Glu189* | p.Glu189* | p.Gln197*; p.Arg200Gln | p.Gln197*; p.Arg200Gln |
|  | **Zygozity** | Homozygous | Homozygous | Homozygous | Homozygous | Homozygous | Homozygous | Homozygous | Homozygous | Homozygous | Compound heterozygous | Compound heterozygous |
| **Neuroimaging** | **White matter abnormalities** | Periventricular, globi pallidi, external capsules, cerebellar peduncles, dentate hilus, pons. | Periventricular, globi pallidi, external capsules, cerebellar peduncles, dentate hilus, pons. | Periventricular, globi pallidi, external capsules, cerebellar peduncles, dentate hilus, pons. | Periventricular, globi pallidi, external capsules, cerebellar peduncles, dentate hilus, pons. | Periventricular, globi pallidi, external capsules | Periventricular, globi pallidi, external capsules | Periventricular, thalamus, cerebellar peduncles | Periventricular, thalamus, cerebellar peduncles | Periventricular, thalamus, cerebellar peduncles | Periventricular, thalamus, cerebellar peduncles | Periventricular, thalamus, cerebellar peduncles |
|  | **Cerebral atrophy** | No | No | No | No | No | No | No | No | No | No | No |
|  | **Basal ganglia abnormalities** | No | No | No | No | No | No | No | No | No | No | No |
|  | **Peri/Paraventricular cysts** | No | No | No | No | No | No | No | No | No | No | No |
|  | **Thin corpus callosum** | No | No | No | Yes | No | No | No | No | No | No | No |
|  | **Cerebellar atrophy** | Yes, vermis and superior cerebellar peduncles | Yes, vermis and superior cerebellar peduncles | Yes, vermis and superior cerebellar peduncles | Yes, vermis and superior cerebellar peduncles | No | No | Yes, cerebellar vermis | Yes, cerebellar vermis | No | No | No |
|  | **Other** | Normal NCS and EMG | Normal NCS and EMG | No | No | No | No | ERG-normal, VEP-delayed  BAEP-delayed | ERG-normal, VEP-delayed  BAEP-delayed | NA | NA | NA |

| **Study** | | **Anazi et al** | **Baldi et al** | | | | | | | | | |
| --- | --- | --- | --- | --- | --- | --- | --- | --- | --- | --- | --- | --- |
| **Family** | | **XIII** | **XIV** | **XV** | **XVI** | | **XVII** | | **XVIII** | **XIX** | | |
| **Subject** | | 23 | 24 | 25 | 26 | 27 | 28 | 29 | 30 | 31 | 32 | 33 |
| **Mutation** | **c.DNA change** | c.196delC | c.196del | c.196del | c.196del | | c.487C>G | | c.487C>G | c.608G>A | | |
|  | **Amino acid change** | p.Arg66Glyfs*122 | p.Arg66Glyfs*122 | p.Arg66Glyfs*122 | p.Arg66Glyfs*122 | | p.Leu163Val | | p.Leu163Val | p.Trp203* | | |
|  | **Zygozity** | Homozygous | Homozygous | Homozygous | Homozygous | | Homozygous | | Homozygous | Homozygous | | |
| **Neuroimaging** | **White matter abnormalities** | Hypomyelination (no further details available) | Diffuse hypomyelination | Diffuse hypomyelination | Diffuse hypomyelination | Diffuse hypomyelination | Diffuse hypomyelination | NA | No | No | Diffuse hypomyelination | Diffuse hypomyelination |
|  | **Cerebral atrophy** | NA | No | NA | Generalized | Generalized | Generalized | Yes | No | No | Yes | Yes |
|  | **Basal ganglia abnormalities** | NA | NA | NA | NA | NA | NA | NA | NA | NA | NA | NA |
|  | **Peri/Paraventricular cysts** | NA | NA | NA | NA | NA | NA | NA | NA | NA | NA | NA |
|  | **Thin corpus callosum** | NA | No | NA | Yes | Yes | Hypoplasia | NA | No | No | Yes | Yes |
|  | **Cerebellar atrophy** | NA | No | NA | NA | NA | No | NA | No | No | Yes (no further details available) | Yes (no further details available) |
|  | **Other** | NA | No | No | No | No | No | No | No | No | No | No |
